# Editorial: mHealth tools for patient empowerment and chronic disease management

**DOI:** 10.3389/fpsyg.2023.1206567

**Published:** 2023-06-14

**Authors:** Pedro Sousa, Ricardo Martinho, Pedro Parreira, Gang Luo

**Affiliations:** ^1^The Health Sciences Research Unit: Nursing (UICISA: E), Nursing School of Coimbra (ESEnfC), Coimbra, Portugal; ^2^Center for Innovative Care and Health Technology (ciTechcare), Polytechnic of Leiria, Leiria, Portugal; ^3^School of Technology and Management, Polytechnic of Leiria, Leiria, Portugal; ^4^CINTESIS-Center for Health Technology and Services Research, University of Porto, Porto, Portugal; ^5^University of Washington, Seattle, WA, United States

**Keywords:** medical devices, eHealth, mHealth, chronic disease, self-management, digital health technology

Modern societies are facing new healthcare challenges with the integration of digital health interventions as a part of their healthcare systems. However, the digital transformation of healthcare requires active patient engagement as a core component of healthcare interventions. In the case of chronic diseases, new digital tools are believed to help maintain and improve patient health and care, by optimizing the course of disease treatment.

Indeed, facilitating access to quality health services and building the capacity to reduce risk are key priorities around the world. Nevertheless, health systems are facing unprecedented financial pressures at a time of growing demand for their services.

Technology can help people use care services less by promoting healthier lives. Prompt advances in wearable sensor technologies and mobile communications could close the gap between home- and clinic-based care delivery models by linking accessibility, availability, and responsive, tailored clinical oversight. Digital health solutions can help democratize access to medical care.

Even though mobile health (mHealth) tools are widely recognized as a promising resource capable of changing healthcare, additional research is needed to enhance knowledge about their limitations and benefits for chronic disease management and patient empowerment.

The major aim of the present Research Topic was to collect new evidence, reviews, clinical experiences, and perspective articles about the current practice for chronic disease management and patient empowerment using digital solutions and mobile technology.

This Research Topic includes 19 articles of different types: 11 original research papers, 3 perspective papers, 3 study protocols, 1 systematic review and 1 methods paper. The collection includes diverse digital solutions, such as conversational agents, medical devices, online healthcare platforms, clinical decision support systems, and mHealth apps, among others. Regarding the chronic conditions addressed by these articles, we may find cardiovascular diseases, diabetes, hypertension, cancer, tuberculosis, rheumatic diseases, Parkinson's, inflammatory bowel disease, and palliative care, among others.

Technology advances are producing innovative tools and resources for patient monitoring and health management, specifically important for chronic conditions. The use of mHealth technologies is raising, and these methods are contributing to assisting both healthcare professionals and patients in education and disease management.

Laranjeira et al. propose a mixed-method study that offers new insights into the expectations and needs of nurses and patient-informal caregivers' dyads in community palliative care on online health resources and caregiving preparedness. With the implementation of an adaptive digital tool, they aim to enhance access to palliative care family support, addressing the lack of accessible and available in-person counseling resources. This Digital Health Intervention may be used for communication, symptom management, decision-making, and education, to improve the informal caregivers' and patients' quality of Life, promoting anticipatory grief and the efficiency of the services.

The ubiquitous usage of mobile phones makes them an adequate platform for delivering interventions to promote awareness and knowledge. In the Yusuf et al. study, the use of a mobile app increased the women's knowledge of breast cancer risk factors, confidence level for breast self-examination and warning signs awareness.

mHealth reminders also become promising approaches to support chronic patients' treatment. Wu et al. evaluated the effect of a reminder app and the smart pillbox on tuberculosis treatment outcomes compared with standard care. They concluded that the reminder app and the smart pillbox interventions were acceptable and improved the treatment outcomes.

Rosa et al. article reviewed some of the mobile apps available to monitor individual chronobiology and lifestyle behaviors. They also described the development of a mHealth solution developed to monitor these variables, the NutriClock system.

Additionally, Yao et al. evaluated the reliability and accuracy of steps tracked by the smartphone-based WeChat app compared with the Actigraph-GT3X accelerometer. Despite the limitation in predicting body composition, they concluded that the app was reliable and could be used to assess physical activity step counts in free-living conditions.

Indeed, there is a raising number of digital resources, including diagnostic decision support systems, to better manage and assess symptoms, provide tailored treatment, and understand where and when to seek medical care. Ventura, Sousa, et al. presents a study protocol to assess the effectiveness of a comprehensive Clinical Decision Support System for remote patient monitoring of cardiovascular disease patients. mHealth tools may contribute to more tailored patient recommendations according to their specific needs.

mHealth tools allow sharing of relevant information and data with physicians and other healthcare professionals, continuous monitoring of patients, and accessing education resources to support informed decisions.

The development of e-health contributed to the growth of online health communities, representing convenient sources of information for patients who have temporal and geographical constraints on visiting physical healthcare institutions. Zhang L. et al. analyzed an online healthcare platform (Spring Rain Doctor), a new form of medical treatment that tried to minimize the unbalanced distribution of medical resources in China. According to the authors, the online healthcare platform has reduced the medical pressure of the hospital and the risk of cross-infection, especially during the COVID-19 pandemic. The total number of platform users is 130 million, and its daily consulting times go beyond 300,000. Additionally, the study by Liu et al. explored how patients' self-disclosure affects the establishment of patients' trust in physicians, analyzing computer-mediated communication in an online health community.

Effective management of chronic conditions requires patients to be motivated to make significant behavior changes to improve wellbeing, quality of life, and health outcomes. Digital solutions may be crucial to achieving these aims, although is still scarce the information about which features increase treatment adherence, which can contribute to behavior change and are more engaging over time.

Kassavou et al. present a post-trial process evaluation of the mechanism by which an e-health intervention was effective at increasing medication adherence in non-adherent patients with Type 2 Diabetes or Hypertension. This mixed methods research found that effectiveness was associated with the primary mechanisms of behavior change and that the intervention supported motivation and ability to adhere.

Also, Ventura, Brovall, et al. considered that digital health solutions became essential complementary solutions in health to improve communication and support at a distance, with evidence of enhancing patient outcomes. Their discussion supports the adoption of mixed-methods studies, to gather the perspectives of end-users and stakeholders, as well as pragmatic evaluation approaches that value effectiveness and process outcomes.

Anthropomorphic conversational agents are other promising digital tools to support the self-management of chronic diseases. Recently, the systematic review of Griffin et al. (2021) showed some evidence that text-based conversational agents (chatbots) are acceptable, usable, and effective in supporting chronic disease self-management. In this Research Topic, Pernencar et al. also undertook a literature review associating the use of chatbot technology with inflammatory bowel disease patients' health care. Chatbot technology for chronic disease self-management increases self-care practice, presenting a huge potential as a part of digital health interventions.

The study of Pimenta et al. describes the development of a theory-based intervention to increase physical activity in older adults with type 2 diabetes, included in a multi-behavior e-health intervention with a chatbot. This study includes several behavior change techniques and may leverage the efforts of others in developing analogous interventions.

Currently, multi-channel appointments have been provided for patients to receive medical care, namely for those with remote distance and severe conditions. Ye and Wu decided to address the long treatment waiting time in the outpatient clinic. This study confirms the effect of Internet use on decreasing patients waiting time and explores the factors that influence patient appointment channel choice, producing several insights into the design of appointment systems in hospitals.

Huang et al. present a novel WeChat Applet to help patients with dental anxiety management, that provides a physical status self-evaluation, online assessment, and teleconsultant. The results of this application show that it is a useful and relevant tool, before and after dental treatment, being effective in improving patients' satisfaction and dentists' convenience and reducing treatment risks, especially regarding the management of high-risk patients during the COVID-19 pandemic.

Chronic disease management is a lifelong process, commonly self-managed by the patient. Artificial Intelligence can help patients improve treatment adherence and continuously monitor their health data (Kent, 2020). Artificial Intelligence also addresses the need for clinicians to make efficient informed decisions by keeping track of several patients at once and directing treatment to those who need it the most (DrKumo, 2022).

Knitza et al. evaluated the usability, usefulness, acceptability, and potential impact of artificial intelligence-based symptom checkers and an online questionnaire-based self-referral tool. Results showed that patients increasingly evaluate their symptoms independently online, nonetheless only few used diagnostic decision support systems or dedicated symptom assessment websites. Decision support systems, such as online questionnaire-based self-referral tools and artificial intelligence-based symptom checkers are easy to use, well accepted among patients with musculoskeletal complaints, and may replace online search engines for patient symptom assessment, increasing helpfulness and saving time.

The study protocol of Bevilacqua et al. aims to evaluate a rehabilitation program for Parkinson's disease patients based on a new system called “SI-ROBOTICS”, composed of multiple technological components, as a dance-based game, a social robotic platform with an artificial vision setting, wearable and environmental sensors. The study proposes a new approach to Parkinson's disease rehabilitation, focused on the use of Irish dancing, including a new technology that helps patients to perform dance steps and collect performance and kinematic parameters.

We may also highlight the importance of smart devices and wearables, that provide patient-centered health data in real-time, helping inform self-management decision-making. Although their perceived benefits in improving chronic disease self-management, their influence on healthcare outcomes continues weakly understood.

Bernardes, Ventura, et al. present a perspective on integrating wearable technology and IoT to support the self-management and telemonitoring of Parkinson's disease patients. Adding current treatment solutions with e-health strategies in Parkinson's disease patients' real-world environments is critical to improving their quality of life. Thus, IoT and wearable technology might constitute resources of excellence in self-management and continuous monitoring in people's home environments.

Bernardes, Caldeira et al. present a study protocol to perform an innovative evaluation of nursing students' podiatric profile (using a pedobarographic platform), which will allow for an extensive description of foot/ankle changes and their relationship with walking contexts and prolonged standing.

Finally, Zhang X. et al. used an electronic portable spirometer (GOSPT2000), a validated device for dynamic monitoring of lung function, to investigate the possible influencing factors of the large- and small-airway function variation in healthy non-smoking adults.

Taken together, these 19 studies indicate the future direction of the field. They show that digital health tools, including digital therapeutics, wearables, remote patient monitoring, coaching, and education, among others, can assist with condition-specific factors. Despite the potential for the delivery of cost-effective healthcare, the implementation of e-health interventions is not an easy effort. It is unreasonable to have a universal “digital recipe” for managing chronic diseases (Bashi et al., 2020). From a patient's perspective, self-management strategies for a chronic disease depend on the economic and socio-cultural status of people. From a health system's perspective, different countries have different policy and legislative implications for the adoption of digital health interventions.

## Author contributions

All authors listed have made a substantial, direct, and intellectual contribution to the work and approved it for publication.
